# Fellow-Workers and Their Doings

**Published:** 1914-03-28

**Authors:** 


### ROUND THE WORLD.

### Fellow-Workers and their Doings.

**[The Editor Invites Early Information and Contributions Marked "Fellow-Workers."]**

Dr. Diarmid Noël Paton, one of the three medical men whose names have been submitted for election as Fellows of the Royal Society, is a leading physiologist and occupies the Chair of Regius Professor of Physiology in Glasgow University, his work on metabolism having greatly advanced our knowledge of that difficult branch of physiology. Before going to Glasgow, Professor Paton was lecturer on physiology in the Edinburgh School of Medicine and superintendent of the research laboratories of the Edinburgh Róyal College of Physicians; he is the author of a well-known text-book on "Human Physiology."

A new treatment for asthma, outlined by Dr. F. G. Crookshank in the *Lancet* (March 14), seems to offer possibilities of relief to many sufferers. This investigator suggests that in pituitary gland extract, some uses of which were referred to in The Hospital recently (March 14), we have a substance which can be employed without danger for the lasting relief of asthmatic paroxysms. Dr. Crookshank, who is physician to out-patients at Hampstead General Hospital and to the Belgrave Hospital for Children, advises adult patients to take pituitary gland substance made up into tablets in doses of two grains, night and morning, but has not so far used the treatment for children; in his opinion there need be no fear lest deleterious effects on arterial walls will be produced by the use of pituitary extract.

The new member of the surgical staff at the Liverpool Samaritan Hospital for Women, Miss M. H. F. Ivens, is a former student of the London School of Medicine for Women, where she had a remarkably distinguished career, taking the University Scholarship and gold medal in obstetric medicine, with honours in medicine and forensic medicine, at the M.B.Lond. examination in 1900. These successes, Miss Ivens, who has been for some time honorary officer for diseases of women at the Liverpool Stanley Hospital, followed up with honours at the B.S.Lond., subsequently taking the very high surgical qualification of M.S.Lond. Her past experience includes appointments at the Royal Free Hospital, the Clapham Maternity Hospital, and the Canning Town Missionary Hospital.

The new director of the Runcorn Research Laboratory (Liverpool School of Tropical Medicine), Dr. B. Blacklock, has had the advantage of studying tropical diseases locally, having spent several years in Africa for the purpose. An M.D. of Edinburgh University and D.P.H. Lond., Dr. Blacklock has for some time been acting director of the important laboratory of which he has now been placed in permanent charge. His researches have included some important work on the life-history of the trypanosomes and other blood parasites responsible for various diseases which are very fatal to both men and animals in hot countries.

Leprosy is still one of the most intractable scourges of the human race, chaulmoogra oil and its refined derivative "anti-leprol" being the only remedies upon which much reliance is placed. Much interest, therefore, attaches to the good results Dr. MacLeod, physician to the skin department at Charing Cross Hospital, is obtaining from the use of injections of a concentrated watery extract of lepra bacilli toxins used similarly to Koch's alt-tuberculin.

Dr. C. E. Zundel, M.D., M.R.C.P., who has been appointed assistant physician to the *Dreadnought* Hospital at Greenwich, received his medical education at the London Hospital, which institution he entered on leaving the Elizabeth College in Guernsey. Having occupied several posts at the "London," Dr. Zundel became particularly interested in the diseases of early life, and was soon appointed physician (out-patient) to the Evelina Hospital for Sick Children.

Dr. J. George Adami, F.R.S., the distinguished recipient of the Fothergillian gold medal (1914) awarded by the Medical Society of London, has been well known for many years to all students of pathology as an investigator whose work never fails to throw fresh light on the problems attacked by him. As Professor of Pathology in the McGill University at Montreal, Dr. Adami occupies an influential position, and his theories of sub-infection, although perhaps not accepted everywhere, have certainly offered a reasonable explanation of the origin of many forms of disease. In his early days Professor Adami was a house surgeon at the Manchester Royal Infirmary and demonstrator of pathology at Cambridge.

Mr. W. H. Hey, M.B., F.R.C.S., who has also been promoted to the post of assistant surgeon to the Manchester Royal Infirmary, qualified M.B., Ch.B. Vict. Man. in 1905. He holds the appointments of honorary surgeon to the Ancoats Hospital, visiting surgeon to the Manchester Children's Hospital, and medical superintendent to the Christie Cancer Hospital.

